# Frontiers in plasma proteome profiling platforms: innovations and applications

**DOI:** 10.1186/s12014-024-09497-2

**Published:** 2024-06-21

**Authors:** Rajesh Kumar Soni

**Affiliations:** 1 Proteomics and Macromolecular Crystallography Shared Resource, Columbia University Irving Medical Center, New York, USA; 2 Department of Pathology and Cell Biology, Columbia University Irving Medical Center, New York, USA

**Keywords:** Biomarker, Biofluids, Secretome, Plasma proteome, Neat plasma, Seer proteograph, ENRICH-iST, timsTOF Pro 2, Proteograph XT

## Abstract

Biomarkers play a crucial role in advancing precision medicine by enabling more targeted and individualized approaches to diagnosis and treatment. Various biofluids, including serum, plasma, cerebrospinal fluid (CSF), saliva, tears, pancreatic cyst fluids, and urine, have been identified as rich sources of potential for the early detection of disease biomarkers in conditions such as cancer, cardiovascular diseases, and neurodegenerative disorders. The analysis of plasma and serum in proteomics research encounters challenges due to their high complexity and the wide dynamic range of protein abundance. These factors impede the sensitivity, coverage, and precision of protein detection when employing mass spectrometry, a widely utilized technology in discovery proteomics. Conventional approaches such as Neat Plasma workflow are inefficient in accurately quantifying low-abundant proteins, including those associated with tissue leakage, immune response molecules, interleukins, cytokines, and interferons. Moreover, the manual nature of the workflow poses a significant hurdle in conducting large cohort studies. In this study, our focus is on comparing workflows for plasma proteomic profiling to establish a methodology that is not only sensitive and reproducible but also applicable for large cohort studies in biomarker discovery. Our investigation revealed that the Proteograph XT workflow outperforms other workflows in terms of plasma proteome depth, quantitative accuracy, and reproducibility while offering complete automation of sample preparation. Notably, Proteograph XT demonstrates versatility by applying it to various types of biofluids. Additionally, the proteins quantified widely cover secretory proteins in peripheral blood, and the pathway analysis enriched with relevant components such as interleukins, tissue necrosis factors, chemokines, and B and T cell receptors provides valuable insights. These proteins, often challenging to quantify in complex biological samples, hold potential as early detection markers for various diseases, thereby contributing to the improvement of patient care quality.

**Supplementary Information:**

The online version contains supplementary material available at 10.1186/s12014-024-09497-2.

## Background

Biomarkers, encompassing measurable substances, structures, or biological processes in the body, play a crucial role in various aspects of medical research and clinical applications. They are integral to disease diagnosis, prognosis, and monitoring, as well as drug development and the emerging field of personalized medicine [1, 2].

Cutting-edge technologies such as genomics, proteomics, metabolomics, and imaging techniques enable the identification and validation of biomarkers. Traditionally, tissue biopsy has been a cornerstone for diagnosis, providing histological and mutational profiles. However, this method is invasive and presents challenges in sample accessibility, repetition frequency, patient comorbidities, tissue storage, and sample integrity maintenance. Overcoming these barriers, various non-invasive biofluids such as blood, urine, saliva, or cerebrospinal fluid serve as rich sources of biomarkers [3]. Nonetheless, the high complexity and large dynamic range of protein abundance in these fluids pose challenges to mass spectrometry analysis, affecting sensitivity, coverage, and precision [4].

Despite the availability of over 100 FDA-approved plasma or serum tests for detecting highly abundant proteins and certain tissue leakage protein biomarkers [5], many of these biomarkers are not available for early disease detection in conditions such as cancer, cardiovascular diseases (CVDs), and neurological disorders. The increasing burden of CVDs, now the leading cause of death in the United States [6, 7], highlights the urgent need for effective biomarkers. Similarly, neurological disorders contribute significantly to global morbidity and mortality [8], underscoring the importance of biomarker research in this field.

Early detection plays a crucial role in mitigating the impact of cancer, the second leading cause of death worldwide [9]. Biomarkers such as cardiac troponin, C-reactive protein, and natriuretic peptides are pivotal in the early detection and prognosis of heart failure [10]. While clinical tests for conditions like ALS and FTD are lacking, recent research has identified abnormal proteins, notably TDP-43 dysfunction, in the spinal fluid of individuals with these conditions [11], offering promise as potential protein biomarkers for improvement.

Advancements in mass spectrometry proteomics technologies, including improvements in sensitivity, scan speed, reproducibility, and dynamic range coverage, make it possible to work with complex biological samples such as biofluids. Automation of sample preparation and high-throughput LC–MS/MS systems enable large-scale clinical studies, addressing the pressing need for early disease detection biomarkers [12]^.^ Improved data analysis pipelines and machine learning tools such as OmicLearn [13] and Clinical Knowledge Graph (CKG) [14] accelerate the analysis and interpretation of large cohort studies, facilitating informed clinical decision-making.

This study evaluates three distinct workflows Neat Plasma, ENRICH-iST, and Proteograph XT for their applicability in biomarker discovery. The assessment focuses on reproducibility, robustness, and the ability to achieve comprehensive proteome coverage, utilizing human pool plasma samples as a representative model. Among these workflows, Proteograph XT stands out as it outperforms others while also offering complete automation of sample preparation, thereby providing a promising avenue for advancing early detection disease biomarker discovery.

## Methods

### Neat plasma sample preparation

1 μL of Neat Plasma samples was diluted at a 1:10 ratio with 100 mM Tris–HCl, pH 8.5. Subsequently, 1.5 μL of the diluted plasma samples were resuspended in 40 μL of freshly prepared SDC lysis buffer [15] (1% SDC and 100 mM Tris–HCl, pH 8.5) and boiled for 15 min at 60 °C, 1200 rpm for denaturation. Protein reduction and alkylation of cysteines were carried out using 10 mM TCEP and 40 mM CAA for 10 min at 45 °C, 1200 rpm followed by sonication in a water bath, cooled down to room temperature. Protein digestion was performed overnight by adding LysC/trypsin mix in a 1:50 ratio (µg of enzyme to µg of protein) at 37 °C and 1400 rpm. The resulting peptides were acidified by adding 1% TFA, vortexed, and subjected to Stage Tip clean-up via SDB-RPS [15], followed by drying in a speed-vac. The peptides were then resuspended in 10 μL of LC buffer (3% ACN/0.1% FA). Peptide concentrations were determined using NanoDrop, and 200 ng of each sample was utilized for diaPASEF analysis on timsTOF Pro 2.

### Plasma sample preparation with ENRICH-iST workflow

Plasma samples were processed using the PreOmics ENRICH-iST Kit following the vendor’s provided protocols [16]. In brief, 20 µL of plasma samples were incubated with pre-washed EN-BEADS for 30 min at 30 °C and 1200 rpm in 1.5 mL Eppendorf tubes on a ThermoMixer with EN-BIND buffer. Proteins bound to EN-BEADS were washed three times, and the proteins were further processed using the iST-BCT workflow, optimized for biofluids. Next, 50 μL of LYSE-BCT was added to each Eppendorf tube, and the samples were heated at 95 °C for 10 min with agitation at 1200 rpm. After cooling the Eppendorf tubes to room temperature, a trypsin digestion buffer was added, and the tubes were incubated at 37 °C for 3 h with shaking at 1200 rpm. The digestion process was stopped by adding the supplied stop buffer, and the remaining reaction supernatant was cleaned up using the provided filter cartridge. The peptides were eluted twice with 100 μL of elution buffer and combined.

The peptides were then dried in a speed-vac and resuspended in 10 μL of LC buffer (3% ACN/0.1% FA). Peptide concentrations were determined using NanoDrop, and 200 ng of each sample was utilized for diaPASEF analysis on the timsTOF Pro 2.

### Plasma sample preparation with Proteograph XT workflow

240 µL plasma samples were used. The corona formation, wash, protein lysis and alkylation, digestion, and peptide cleanup were done on Proteograph XT workflow on SP100 Automation Instrument (Seer) as described [17]. The eluted peptides were dried in a speed-vac and resuspended in 10 μL of LC buffer (3% ACN/0.1% FA). Peptide concentrations were determined using NanoDrop, and 200 ng of each sample was utilized for diaPASEF analysis on the timsTOF Pro 2.

### Liquid chromatography with tandem mass spectrometry (LC–MS/MS)

Peptides were separated over 65 min at a flow rate of 300 nL/min using a reversed-phase C18 column with an integrated CaptiveSpray Emitter (25 cm × 75 µm, 1.6 µm, IonOpticks). Mobile phases A and B consisted of 0.1% formic acid in water and 0.1% formic acid in acetonitrile, respectively. The percentage of mobile phase B was increased linearly from 2 to 25% over 35 min, followed by a further increase to 40% over 10 min, then to 95% over 10 min, and finally, the column was washed for 10 min. The timsTOF Pro 2 operated in diaPASEF mode [18] with data acquired across defined 50 Th isolation windows from 350 to 1000 m/z and 0.66 to 1.31 1/K0 for mass and IM range, respectively. To adjust the MS1 cycle time in diaPASEF, repetitions were set to 2 in the 13-scan diaPASEF scheme. The collision energy was ramped linearly as a function of mobility from 59 eV at 1/K0 = 1.60 Vs cm^−2^ to 20 eV at 1/K0 = 0.60 Vs cm^−2^. Detailed LC–MS/MS settings are provided in the Supplementary Information, Table S3.

### Data analysis

The acquired diaPASEF raw files were searched using the UniProtKB/Swiss-Prot Homo sapiens database (downloaded in 2023, 42,356 entries) was performed using library-free workflow in the DIA- DIA-NN 1.8.1 [19] search engine, employing the default settings of the library-free search algorithm with match-between-runs (MBR) enabled. A maximum of 1 trypsin missed cleavage was allowed and the maximum variable modification was set to 1. Carbamidomethylation was set as the fixed modification, whereas protein N-terminal methionine excision, methionine oxidation, and N-terminal acetylation were set as variable modifications. The peptide length range was set to 7–30 amino acids, precursor charge range 1–4, precursor m/z range 300–1000, and fragment ion m/z range 200–1800. The false discovery rates (FDRs) at the protein and peptide levels were set to 1%, and MS1 and MS2 mass tolerances were automatically determined by DIA-NN.

Results obtained from DIA-NN were subjected to further statistical analyses, and data visualizations were performed using R software version 4.2.3 and RStudio version 2023.12.0 + 369. All plots, including bar plots, protein rankings, and coefficients of variation (CV), were created using the ggplot2 library from the tidyverse package. The Venn diagram was generated using a web-based tool (https://bioinformatics.psb.ugent.be/webtools/Venn/), and gene ontology (GO) term and pathway analyses were performed using the R package clusterProfiler [20]. Heatmaps were created using the statistical tool Perseus (1.6.15.0) (from MaxQuant) [21]. All R scripts and plots generated to compose the figures are reported on are provided in Supplementary Material by figure number.

## Results

### A comprehensive comparison of workflows for plasma proteome profiling

**Fig. 1:**
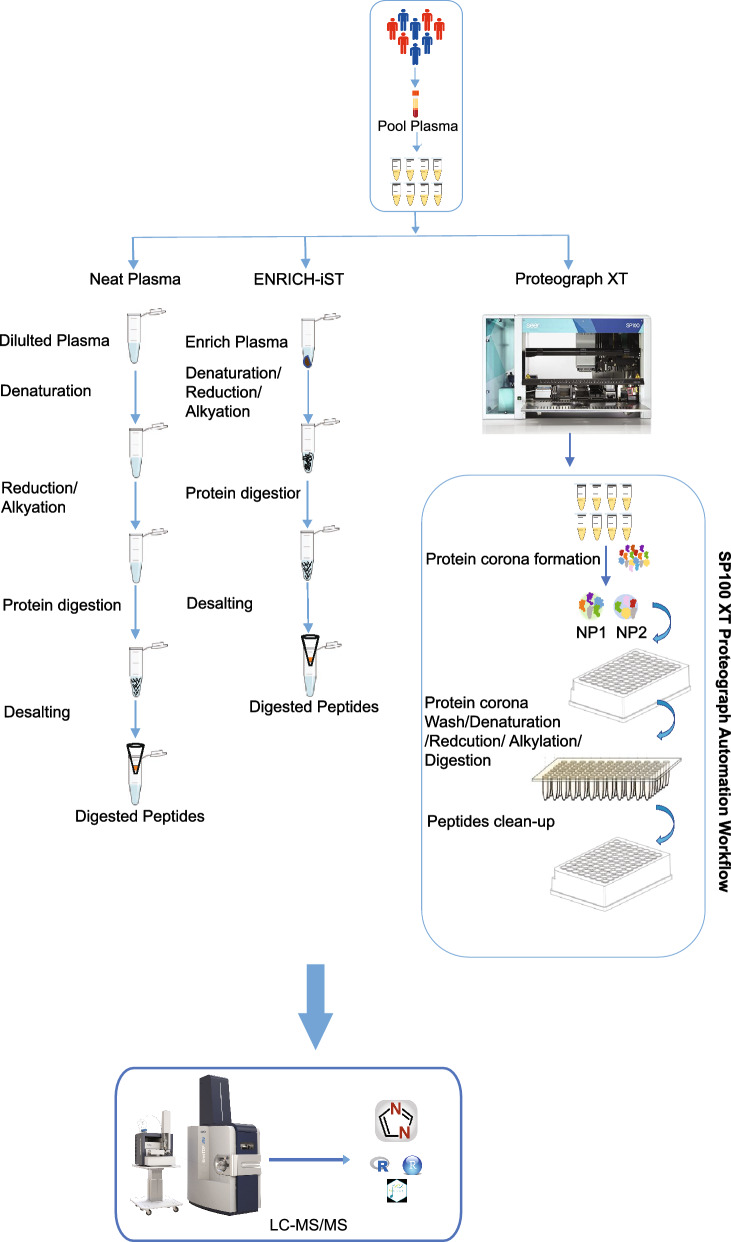
Plasma sample preparation workflows. A visual representation comparing the Neat Plasma, ENRICH-iST, and Proteograph XT workflows step by step.

Early disease detection relies on the identification and quantification of reliable biomarkers. The pooled human plasma samples were divided into 8 aliquots, and each aliquot underwent processing to evaluate the Neat Plasma workflow and commercially available sample preparation kit ENRICH-iST and the fully automated Proteograph XT workflow, as depicted in (Fig. 1). The Neat Plasma workflow entails manual processing with laboratory reagents, while the ENRICH-iST approach enriches proteins, providing a streamlined sample preparation workflow. In contrast, the Proteograph XT workflow is fully automated and utilizes two nanoparticles, selectively enriching an unbiased subset of proteins in complex plasma samples.

**Fig. 2:**
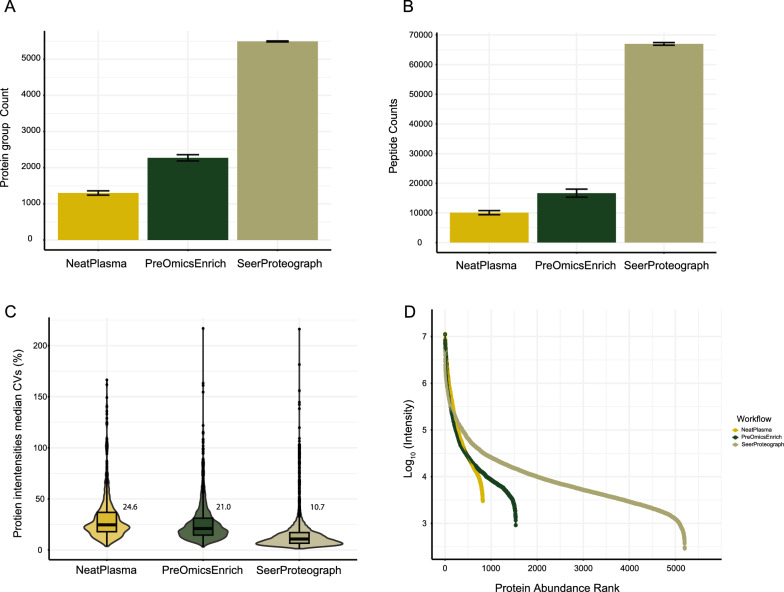
Plasma sample preparation workflow comparison. **A **Protein groups identified by each workflow **B** Peptides identified by each workflow. **C** Coefficient of Variation (CV) for median quantified protein intensities within workflows. **D** The dynamic range of quantified protein abundance across workflows

All three workflows (Fig. 1) were processed using identical pooled plasma aliquots, and data acquisition was conducted on a timsTOF Pro 2 instrument with a 65 min gradient and diaPASEF method. The subsequent data analysis was performed employing DIA-NN. Initially, the protein identification performance of each workflow was assessed. Across all three workflows, approximately 5881 protein groups were identified. Notably, Proteograph XT exhibited superior performance, identifying, and quantifying over 4.2-fold more protein groups compared to Neat Plasma and 2.4-fold more compared to ENRICH-iST (Supplementary Information: Table S1, Fig. 2A). Similarly, 66,987 peptides were identified, with Proteograph XT quantifying over 6.7-fold more compared to Neat Plasma and fourfold more compared to ENRICH-iST (Supplementary Information: Table S2, Fig. 2B).

The protein dynamic range and complexity play crucial roles in the depth of the quantified plasma proteome, with Neat Plasma samples providing the least information. However, ENRICH-iST exhibits improvement compared to Neat Plasma, and the Proteograph XT workflow outperforms both alternatives.

Large cohort studies rely on a robust and reproducible workflow. We compared the quantified normalized intensity of protein groups within different workflows Neat Plasma, ENRICH-iST, and Proteograph XT. The Neat Plasma, ENRICH-iST, and Proteograph XT workflows yielded a median coefficient of variation (CV) of 24.6, 21.0, and 10.7%, respectively, as shown in (Fig. 2C). The Proteograph XT workflow demonstrated the lowest CV compared to the Neat Plasma and ENRICH-iST workflows, attributed to the uniform and consistent enrichment of proteins using SeerProteograph's nanoparticle technology, operating across a large dynamic range. The fully automated capabilities of SeerProteograph also contribute to minimizing technical challenges in the workflow.

Plasma/serum samples are complex due to the broad dynamic range of proteins, posing challenges for the identification and quantification of low-abundant proteins through LC–MS/MS. To assess the dynamic range covered by each workflow, we utilized a protein abundance ranking of protein groups' normalized intensities, revealing an approximate span of 4.6 orders of magnitude. The Proteograph XT workflow significantly increased the number of quantified proteins by over 6.3-fold and 3.4-fold compared to the Neat Plasma and ENRICH-iST workflows. This extension indicates a highly efficient reduction of the dynamic range (Fig. 2D) compared to the Neat Plasma and ENRICH-iST workflows.

### Comparative analysis of workflows for secretome database coverage

Next, we explored the coverage of the secretome database, which comprises soluble proteins and secreted extracellular vesicles, encompassing biologically active factors such as cytokines, interleukins, interferons, chemokines, complement and coagulation factors, hormones, growth factors, enzymes [22]. These proteins, shed from cells/tumors, play a crucial role in cell signaling, communication, and growth, and their abundance changes under various pathological conditions. While these proteins are secreted into the extracellular space, they are generally more abundant in biological fluids [23]. The dynamic nature of secretome protein composition makes them a valuable source of potential biomarkers for cancer and other diseases, aiding in diagnosis, prognosis, and therapeutic monitoring [24].

**Fig. 3:**
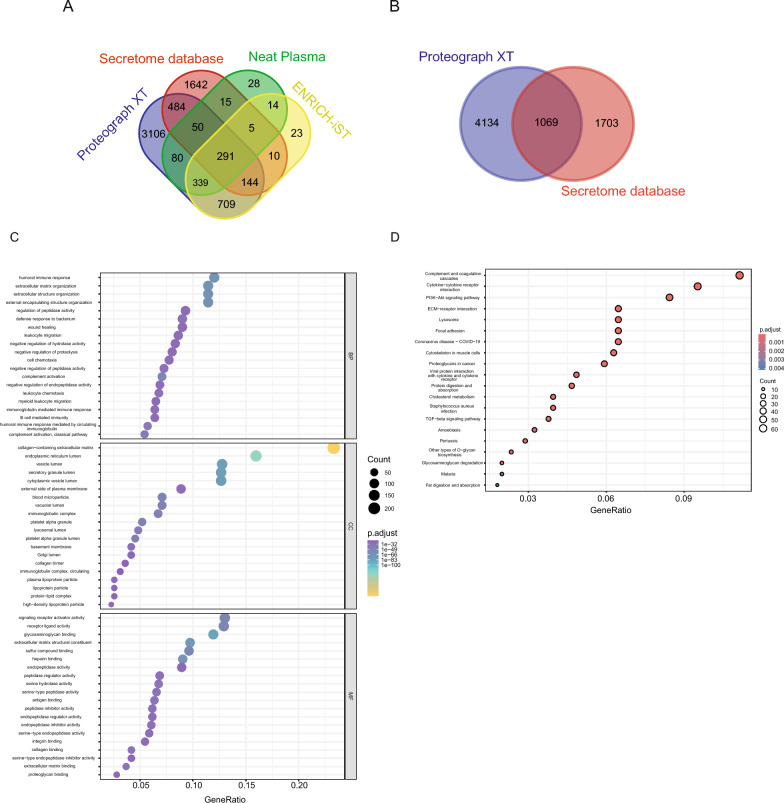
Secretome Protein Database Coverage. **A** Evaluation of protein coverage from the Secretome protein database across workflows. **B** Assessment of the percentage overlap between SeerProteograph and Secretome database protein groups. **C** Gene Ontology (GO) enrichment, and **D** KEEG pathway analysis for protein groups overlapping between Proteograph XT and Secretome database.

The Secretome database, sourced from The Human Protein Atlas [25], underwent a comprehensive comparison across the Neat Plasma, ENRICH-iST, and Proteograph XT workflows to assess coverage. Proteins quantified in all samples within these workflows were included in the analysis, revealing that the Proteograph XT workflow exhibited notably high coverage, particularly in the quantification of low-abundant proteins (Fig. 3A).

For Gene Ontology (GO) terms functional analysis, a ~ 39% overlap of proteins of Proteograph XT workflow was chosen (Fig. 3B). This analysis encompassed Molecular Function (MF), Biological Processes (BP), and Cellular Compartments (CC) (Fig. 3C). The proteins predicted to be secreted into human blood encompassed a diverse array, including well-characterized proteins associated with the extracellular matrix organization, enzymes, receptors, cytokines, complement activation, peptidase activator, humoral immune response, wound healing, leukocyte migration, cell chemotaxis, myeloid leukocyte migration, transport proteins, developmental proteins, defense proteins, enzymes, enzyme inhibitors, integrin binding, antigen binding, glycosaminoglycan binding, collagen binding, B cell-mediated immunity-related proteins, and classical pathway.

While the identified proteins were found in plasma, statistical analysis suggests they are secreted from various cellular compartments, including the endoplasmic reticulum (ER) lumen, vesicle lumen, secretory granule lumen, blood microparticles, lysosomal lumen, platelet alpha granule lumen, Golgi lumen, plasma lipoprotein particles, and protein-lipid complexes.

In the KEGG pathway analysis, these proteins showed significant enrichment for a variety of pathways including complement and coagulation cascades, cytokine-cytokine receptor interaction, PI3K-AKT signaling pathways, ECM-receptor interaction, lysosome, protein digestion and absorption, cholesterol metabolism, TGF-beta signaling pathway, antigen processing and presentation, fat digestion and absorption, glycosaminoglycan degradation pathways (Fig. 3D).

### Comparative analysis of workflows for functional annotation coverage

**Fig. 4:**
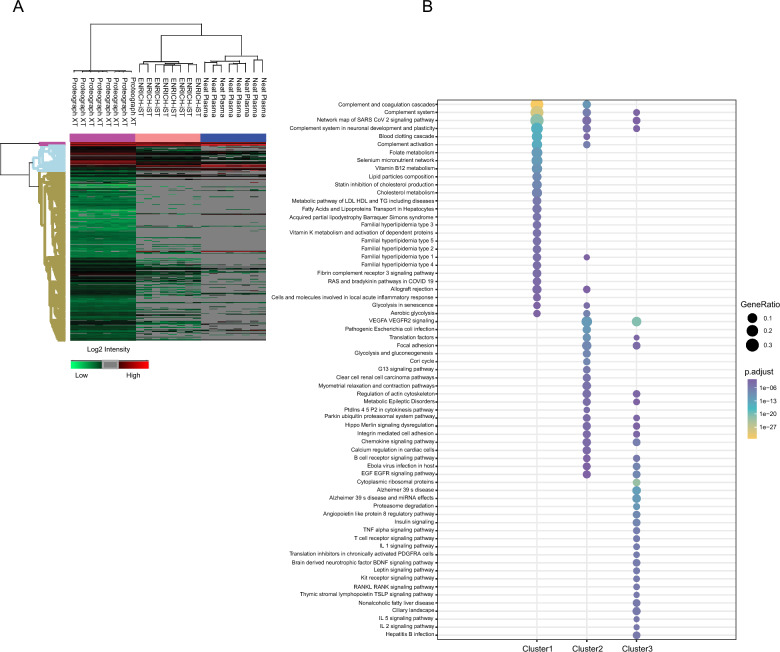
Functional Annotation Coverage. **A **Hierarchical clustering of normalized Log2 protein intensities **B** Pathway analysis of enriched proteins in each identified cluster using the ClusterProfiler package

We investigated the coverage of proteins quantified in three workflows using functional annotation enrichment analysis. Hierarchical clustering of quantified proteins based on their log2 intensity yielded three distinct groups of clusters (Fig. 4A). Each cluster was analyzed for enriched pathways using ClusterProfiler R package of the function of compareCluster with WikiPathways [20] using a threshold of Benjamini and Hochberg (BH) adjusted p-value < 0.05. Proteins covered with cluster 1 showed significant enrichment for a variety of pathways including complement and coagulation cascades, complement system, complement activation, blood clotting cascade, lipid particle composition, cholesterol metabolism, metabolism of triglycerides, and acute inflammatory response. Proteins present in Cluster 1, quantified in all three workflows, these proteins are highly abundant and consistently quantified.

Proteins associated with EGF EGFR signaling, VEGFA VEGFR2 signaling, glycolysis and gluconeogenesis, chemokine signaling pathway, and B cell receptor signaling pathway are enriched by cluster 2. Proteins present in Cluster 2, quantified in ENRICH-iST, and Proteograph XT workflows.

Proteins associated with Insulin signaling, TNF alpha signaling pathway, T and B cell receptor signaling, IL1/2/5 signaling, proteasome degradation pathways were enriched by cluster 3. Cluster 3 proteins were identified in Proteograph XT workflow only, these proteins are low abundant in the samples and could potentially serve as crucial biomarkers.

## Discussion

The emergence of cutting-edge technologies for discovery-based quantitative proteomics, such as ultra-sensitive and high-speed mass spectrometers, fully automated sample preparation systems, and machine learning algorithms for data analysis and quantification, has made it feasible to conduct large cohort studies for novel early diseases biomarker discovery [26, 27].

In this study, various workflows for plasma proteomic profiling were compared to establish a methodology characterized by sensitivity, reproducibility, and depth. Our results demonstrated that the Proteograph XT outperformed other methods, identifying, and quantifying over 4.2-fold more protein groups compared to Neat Plasma and 2.4-fold more compared to ENRICH-iST. Similarly, peptides were identified at a higher rate, with Proteograph XT quantifying over 6.7-fold more compared to Neat Plasma and fourfold more compared to ENRICH-iST. The Proteograph XT workflow's full automation improved the median coefficients of variation (CV) to 10.7%, compared to 24.6% for Neat Plasma and 21.0% for ENRICH-iST. The enhanced depth and dynamic range reduction achieved by the Proteograph XT workflow are crucial for detecting extremely low-abundance proteins. Functional analysis using Gene Ontology (GO) terms and pathway analysis revealed associations with receptors, cytokines, interleukins, immune responses, TNF alpha signaling pathway, and T and B cell receptor signaling, suggesting the potential of these proteins as critical biomarkers.

Each workflow has its limitations. The Neat Plasma workflow, widely used and requiring only 1 µL of sample volume, is inefficient in accurately quantifying low-abundance proteins. Furthermore, its manual nature poses significant hurdles for conducting large cohort studies. Similarly, the ENRICH-iST workflow, although it simplifies the sample preparation process through a vendor-supplied protocol, shares similar limitations in detecting low-abundance proteins and lacks complete automation. This limitation can introduce variability and limit throughput. Moreover, the Proteograph XT workflow demands a larger sample volume of 240 µL of plasma per sample, which may not always be feasible, particularly in studies with limited sample availability. These limitations underscore the urgent need for ongoing improvements in proteomics workflows to enhance their efficiency, automation, and ability to detect low-abundance proteins effectively, which are crucial for advancing discovery in proteomics research.

In conclusion, despite these limitations, the Proteograph XT workflow stands out for its sensitivity, reproducibility, and capacity to provide deep proteome coverage with state-of-the-art mass spectrometers. It holds promise in contributing significantly to the discovery of novel early disease biomarkers through large cohort studies across various diseases such as cancer, neurological disorders, and cardiovascular conditions.

### Supplementary Information

Supplementary material 1.

Supplementary material 2. Table S1_PlasmaProteome_TimsTofPro2_diaPASEF_DIA_NN_ProteinGroups

Supplementary material 3. Table S2_PlasmaProteome_TimsTofPro2_diaPASEF_DIA_NN_Peptides

Supplementary material 4. Table S3_LC_MS_MS Settings

Supplementary material 5. Table S4_ProteinGroup_long_format.csv

Supplementary material 6. Table S5_Peptide_long_format.csv

Supplementary material 7. Table S6_CommonProtein_Seer_Secretomedatabase.csv

Supplementary material 8. Table S7_CommonProtein_Secretome_venn

Supplementary material 9. Table S8_Heatmap_Cluster_1.csv

Supplementary material 10. Table S9_Heatmap_Cluster_2.csv

Supplementary material 11. Table S10_Heatmap_Cluster_3.csv

Supplementary material 12.

## Data Availability

The raw data (diaPASEF) files have been uploaded to Pride and are accessible in Pride PXD050425. Raw proteomics data, statistical analysis, and R scripts used to produce the figures in the manuscript are included in the supplementary information. Any additional information needed for the reanalysis of the data presented in this study is obtainable from the lead contact upon request.

## Declarations

### Generative AI and AI-assisted technologies in the writing process

The author utilized the ChatGPT 3.5 service to enhance English sentences while preparing this manuscript. Following the use of the service, the author thoroughly reviewed and edited the content as needed, taking full responsibility for the publication’s content.
